# No detectable differential microRNA expression between non-atherosclerotic arteries of type 2 diabetic patients (treated or untreated with metformin) and non-diabetic patients

**DOI:** 10.1186/s12933-018-0715-y

**Published:** 2018-05-17

**Authors:** Lasse Bach Steffensen, Søren Feddersen, Simone Rørdam Preil, Lars Melholt Rasmussen

**Affiliations:** 1 Department of Clinical Biochemistry and Pharmacology, Odense University Hospital, Odense, Denmark; 2 Centre for Individualized Medicine in Arterial Diseases (CIMA), Odense University Hospital, Sdr. Boulevard 29, 5000 Odense C, Denmark; 3 Department of Clinical Research, University of Southern Denmark, Odense, Denmark; 4 Department of Cardiovascular and Renal Research, Institute of Molecular Medicine, University of Southern Denmark, Odense, Denmark

**Keywords:** MicroRNA, Diabetes, Artery, Microarray

## Abstract

**Background:**

Type 2 diabetes mellitus (T2DM) is an independent risk factor of cardiovascular disease (CVD), however, the underlying mechanisms are largely unknown. Using non-atherosclerotic internal thoracic arteries (ITAs) obtained from coronary artery bypass grafting, we previously identified a distinct elevation in the level of proteins comprising the arterial basement membrane in T2DM patients not treated with metformin. Altered transcription of genes encoding these proteins has not been observed, indicating alternative mechanisms of dysregulation.

**Methods:**

In this study we screened for differential expression of arterial microRNAs (miRNAs) in T2DM patients to test the hypothesis that the arterial protein signature of diabetic patients is associated with dysregulation at the miRNA level, and further to lay the foundation for novel hypotheses addressing the increased CVD risk of T2DM patients. MiRNA isolated from fresh frozen ITAs [from 18 T2DM- (10 of which were subject to metformin treatment) and 30 non-diabetes mellitus (non-DM) patients] were analyzed by microarray, and miRNAs isolated from formalin-fixated paraffin-embedded (FFPE) ITAs were analyzed by quantitative PCR (qPCR) in an independent study group [26 T2DM- (15 of which were subject to metformin treatment) and 26 non-DM patients] to determine expression levels of miRNAs in a pre-defined panel of 12 miRNAs.

**Results:**

Unexpectedly, no miRNAs were found to be affected by T2DM status in either of the two study groups.

**Conclusions:**

Our data suggest that alternatives to microRNA dysregulation underlie T2DM-associated protein changes in non-atherosclerotic arteries.

**Electronic supplementary material:**

The online version of this article (10.1186/s12933-018-0715-y) contains supplementary material, which is available to authorized users.

## Background

Diabetic patients have increased morbidity and mortality of cardiovascular disease (CVD) including stroke and acute myocardial infarction [1]. The underlying mechanisms are far from understood, however, CVD risk in diabetic patients is independent of hyperlipidemia and hypertension [2] suggesting a direct effect of hyperglycemia and/or insulin resistance within macrovascular tissue. This idea is supported by studies showing that diabetic patients have altered remodeling capacity [3, 4] as well as an augmented prevalence of artery calcification [5] and -stiffening [6]. A paradoxical protection against aneurysms among diabetic individuals is likewise compatible with the notion that specific arterial alterations form the background for cardiovascular consequences of diabetes [7]. Our group previously provided additional support for a direct effect of diabetes in arterial tissue by demonstrating that a distinct group of proteins constituting the vascular basement membrane (including the α1- and α2-chains of collagen IV, as well as ɣ1-laminin and β2-laminin) was elevated in arteries from diabetic patients (40 and 20% for the α1- and α2-chains of collagen IV, respectively) [8]. Diabetic patients treated with metformin, a standard drug with beneficial effects on glucose metabolism and diabetes-related complications [9], had significantly reduced levels of these proteins as compared to non-metformin treated diabetic patients [8]. Interestingly, these protein alterations do not appear to be accompanied by changes of the corresponding gene transcripts [10, 11]. This indicates alternative mechanisms of the observed diabetes-induced effects at the protein level, such as reduced protein degradation (possibly as a consequence of protein glycation or oxidative modifications) and/or altered expression of epigenetic regulators of messenger RNA (mRNA) translation. Especially the idea of microRNA (miRNA)-mediated epigenetic regulation is appealing since *COL4A1* and *COL4A2* mRNA (encoding the α1- and α2-chain of collagen IV, respectively) are subject to pronounced regulation by miRNAs miR-29a and miR-29b [12–17], which are expressed in vascular tissue [18].

MiRNAs are short (~ 22 nucleotides) single-stranded non-coding RNA molecules that bind to target mRNA mainly in 3′ untranslated regions causing translation blockade or mRNA cleavage thereby resulting in decreased translation [19]. Only the former would be detectable by transcriptomic approaches.

Studies investigating miRNA expression in vascular tissue are scant, and to our knowledge, an explorative approach has ever been used to assess the effect of type 2 diabetes mellitus (T2DM) on miRNA expression in non-lesional arteries. We therefore sought to screen for differentially expressed miRNAs by microarray analysis to test the hypothesis that the altered protein signature found in T2DM patients is associated with dysregulation at the miRNA level, and further to lay the foundation for novel hypotheses addressing the increased CVD risk of T2DM patients.

## Methods

### Artery tissue

The Odense Artery Biobank comprises internal thoracic arteries (ITAs) collected from patients undergoing coronary artery by-pass grafting (CABG) at Odense University Hospital in Denmark since 2008. These arteries have been the foundation for several studies addressing vascular pathophysiology [11, 20–24].

### RNA purification

RNA for microarray analysis was isolated from fresh frozen ITAs (from 18 T2DM- (10 of which were subject to metformin treatment) and 30 non-diabetes mellitus (non-DM) patients) using NORGEN Total RNA Purification Kit (#17200,37500, Norgen Biotek Corporation), while RNA for quantitative polymerase chain reaction (qPCR) analysis was isolated from formalin-fixated paraffin-embedded (FFPE) ITAs [from 26 T2DM- (15 of which were subject to metformin treatment) and 26 non-DM patients; four sections of 20 µm from each patient] using NORGEN FFPE RNA Purification kit (#25300, Norgen Biotek Corporation). FFPE sections was obtained from FFPE tissue blocks previously used to determine differential basement membrane protein expression [8]. To allow for normalization of sample-to-sample variation in RNA isolation from FFPE sections and reverse transcription (RT) efficiency, 5 fmol synthetic Arabidopsis thaliana miR-159a (ath-miR-159a) was added to each sample along with buffer RL when using the NORGEN FFPE RNA Purification kit.

### MicroRNA microarray

All experiments and analyses were conducted at Exiqon Services, Denmark. Total RNA was quantified via absorbance spectrophotometry in a NanoDrop 8000 (Thermo Scientific, Wilmington, DE, USA) and verified by an Agilent 2100 Bioanalyzer profile. 750 ng total RNA from sample and reference was labeled with Hy3™ and Hy5™ fluorescent label, respectively, using the miRCURY LNA™ microRNA Hi-Power Labeling Kit, Hy3™/Hy5™ (Exiqon, Denmark). The Hy3™-labeled samples and a Hy5™-labeled reference RNA sample were mixed pair-wise and hybridized to the miRCURY LNA™ microRNA Array 7th Gen (Exiqon, Denmark), which contains capture probes targeting all microRNAs for human (2042 miRNAs), mouse (1281 miRNAs) and rat (723 miRNAs) registered in the miRBASE 18.0. The hybridization was performed according to the miRCURY LNA™ microRNA Array instruction manual using a Tecan HS4800TM hybridization station (Tecan, Austria). One slide cracked in the hybridization station so no profiling data was obtained for this sample (resulting in 48 successful samples). After hybridization the microarray slides were scanned and stored in an ozone free environment (ozone level below 2.0 ppb) in order to prevent potential bleaching of the fluorescent dyes. The miRCURY LNA™ microRNA Array slides were scanned using the Agilent G2565BA Microarray Scanner System (Agilent Technologies, Inc., USA) and the image analysis was carried out using the ImaGene 9.0 software (BioDiscovery, Inc., USA). The quantified signals were background corrected (Normexp with offset value 10 [25]) and normalized using quantile normalization method, which was found to produce the best between-slide normalization to minimize the intensity-dependent differences between the samples. The threshold for detection was calculated for each individual microarray slide as 1.2 times the 25th percentile of the overall signal intensity of the slide. MiRNAs with intensities above threshold in less than 20% of the samples were removed from the final dataset. By this filtering procedure, 677 probes were included in the expression analysis.

In Additional file 1, non-annotated human miRPlus miRNAs are predicted miRNA sequences derived from Exiqon’s database of proprietary material, database mining, and publications.

### Quantitative polymerase chain reaction

12 miRNAs were selected (Table 2) for quantification in FFPE ITA samples. Total RNA was quantified via absorbance spectrophotometry in a NanoDrop 8000 (Thermo Scientific, Wilmington, DE, USA) and miRNA expression was measured by qPCR using TaqMan microRNA assays (Life Technologies). Total RNA was converted to cDNA using the TaqMan MicroRNA Reverse Transcription Kit and Megaplex stem-loop RT primers for Human Pool A and B (Life Technologies) according to the manufacturer’s instructions for low sample input (LSI). For each sample an equal amount of RNA (12 ng) was used as input for each RT and two RT reactions were established; one with Pool A and one with Pool B Megaplex RT primers. Each RT reaction had a final volume of 7.5 µL.

Pre-amplification was performed using Megaplex PreAmp primers for Human Pool A or Pool B (Life Technologies) and TaqMan PreAmp Mastermix (Life Technologies) according to the manufacturer’s instructions for LSI. PreAmp reactions had a final volume of 25 µL and contained 2.5 µL RT-product. Thermal cycling conditions were as follows: 95 °C for 10 min, 55 °C for 2 min, and 72 °C for 2 min, followed by 12 cycles of 95 °C for 15 s and 60 °C for 4 min. Final inactivation was performed at 99.9 °C for 10 min. PreAmp-products were diluted 1:10 in 0.1× TE buffer (pH 8.0).

For the qPCR step 1 µL of diluted PreAmp-product was mixed with TaqMan Universal PCR Master Mix II (2×) and TaqMan miRNA assay (20×) in a final volume of 10 µL. qPCR was performed on a ViiA7 real-time instrument (Applied Biosystems, Foster City, CA, USA) and samples were run in triplicate. Thermal cycling conditions were as follows: 95 °C for 10 min followed by 40 cycles of 95 °C for 15 s and 60 °C for 1 min.

The ViiA7 Real-Time qPCR Analysis Software was used to obtain quantification cycle (Cq)-values, which were exported to the qBase^PLUS^ software (Biogazelle, NV, Belgium) for relative quantification. Cq-values were converted to relative quantities using the method of qBase^PLUS^, which is a modification of the classic delta–delta-Cq method [26]. The modification takes multiple reference genes and gene specific amplification efficiencies into account, as well as the error on the estimated amplification efficiency [26]. Briefly, Cq-values are first converted into relative quantities based on the gene specific amplification efficiency. Normalization is then performed by dividing the relative quantities by a sample specific normalization factor, which is calculated by taking the geometric mean of the relative quantities of the reference genes as described by Hellemans et al. [26].

In order to measure miRNA levels samples were spread across two different runs for each miRNA and data was corrected for inter-run variation using inter-run calibration. For each measured miRNA two samples (inter-run calibrators) were measured in both of the different runs, in addition to the other samples that were spread across the runs. In the qBase^PLUS^ software the results for the inter-run calibrators were used to quantify and correct for inter-run variation by determine a calibration factor for every miRNA-run combination [26]. Normalization was performed using equal amounts of total RNA for each sample and by normalizing to the synthetic miRNA ath-miR-159a using the qBase^PLUS^ software [26]. In addition miRNA data was also normalized using reference genes. First, putative reference genes were evaluated using geNorm [27] as an implementation of the qBase^PLUS^ software. Three putative reference genes for normalization were evaluated for both Pool A and Pool B miRNA data using geNorm. Reference genes were selected based on the average expression stability value M. Based on this, two miRNAs (hsa-miR-103a-3p and hsa-miR-27a-3p) were selected as reference genes for Pool A miRNA data, while one miRNA (hsa-miR-942-5p) and one small RNA (U6 rRNA) were selected as reference genes for Pool B miRNA data. Pool A and Pool B miRNA data were normalized separately. When using endogenous controls for normalization of the miRNA data all P-values given in Table 2 were above 0.05 (data not shown). Hence, fine consistency exist between data normalized using two different normalization strategies.

### Statistics

For patient characteristics, *Student*’s t-tests or *Fisher’s exact* tests were performed when appropriate.

For explorative analysis, *Student*’s t-tests were performed for each of the 677 probes in each of the three two-group comparisons (T2DM patients vs. non-DM patients; non-metformin treated T2DM patients vs. non-DM patients; metformin treated T2DM patients vs. non-metformin treated T2DM patients) followed by *false discovery rate* (FDR) correction of multiple testing.

For the targeted analysis, *Student*’s t-tests were used to test for differential expression in the three two-group comparisons. Intergroup differences accompanied by Bonferroni-corrected P-values below 0.004 [0.05/12 (12 being the number of tests performed)] were considered statistically significant.

## Results

### Tissue and study subjects

**Fig. 1:**
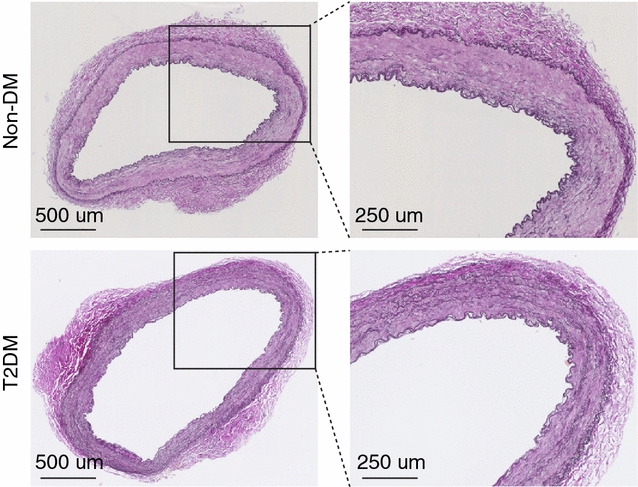
Representative internal thoracic arteries (ITAs) from T2DM and non-DM patients. Weigert stained cross-sections of internal thoracic arteries (ITAs) show lesion-free, homogenous arteries, which are indistinguishable between T2DM and non-DM patients

One hundred ITAs systematically collected in the Odense Artery Biobank over a 9-year period from CABG were analyzed in this study. The ITAs used in this study were all free of histologically apparent lesions, i.e. intimal thickening and atherosclerosis. Although morphologically indistinguishable between T2DM and non-DM patients (Fig. 1), several molecular differences have been identified between these groups within this tissue [8, 10, 11].

T2DM and non-DM patients used in this study were matched on age at surgery, sex, plasma cholesterol, plasma creatinine, blood pressure and statin treatment. T2DM and non-DM patients in study group 1 (microarray/explorative analysis) differed on glycated hemoglobin (HbA1c) and body mass index (BMI), while in study group 2 (qPCR/targeted analysis), these groups differed on HbA1c, high-density lipoprotein (HDL) and triglycerides (TAG) (Table 1).

**Table 1:** Clinical characteristics for each group study

	Study group 1 (explorative analysis)								
	T2DM *n* = 18	Non-DM *n* = 30	P	T2DM (− Met) *n* = 8	Non-DM *n* = 30	P	T2DM (+ Met) *n* = 10	T2DM (− Met) *n* = 8	P
	Mean ± SD	Mean ± SD		Mean ± SD	Mean ± SD		Mean ± SD	Mean ± SD	
Age at surgery, years	66.61 ± 7.51	63.60 ± 8.98	0.219	67.13 ± 8.63	63.60 ± 8.98	0.330	66.20 ± 6.94	67.13 ± 8.63	0.809
HbA1c, %	6.94 ± 1.21	5.74 ± 0.38	0.002	7.37 ± 1.52	5.74 ± 0.38	0.029	6.56 ± 0.76	7.37 ± 1.52	0.236
HbA1c, mmol/mol	52.28 ± 13.07	39.21 ± 4.08	0.002	56.93 ± 16.41	39.21 ± 4.08	0.029	48.21 ± 8.36	56.93 ± 16.41	0.238
Cholesterol, mmol/L	4.19 ± 1.11	4.45 ± 1.16	0.486	4.27 ± 1.16	4.45 ± 1.16	0.721	4.11 ± 1.14	4.27 ± 1.16	0.803
LDL, mmol/L	2.31 ± 0.70	2.61 ± 1.01	0.267	2.41 ± 0.76	2.61 ± 1.01	0.571	2.21 ± 0.68	2.41 ± 0.76	0.614
HDL, mmol/L	1.16 ± 0.38	1.21 ± 0.33	0.684	1.01 ± 0.24	1.21 ± 0.33	0.101	1.30 ± 0.46	1.01 ± 0.24	0.168
Triglycerides, mmol/L	1.88 ± 1.21	1.45 ± 0.63	0.210	2.23 ± 1.54	1.45 ± 0.63	0.234	1.58 ± 0.81	2.23 ± 1.54	0.344
Creatinine, µmol/L	83.72 ± 15.42	85.70 ± 13.14	0.653	76.88 ± 11.22	85.70 ± 13.14	0.080	89.20 ± 16.62	76.88 ± 11.22	0.080
BMI, kg/m^2^	29.98 ± 3.06	27.26 ± 3.77	0.010	29.24 ± 2.57	27.26 ± 3.77	0.104	30.57 ± 3.42	29.24 ± 2.57	0.360
SBP, mm Hg	134.22 ± 19.47	138.42 ± 22.13	0.510	141.38 ± 22.36	138.42 ± 22.13	0.749	128.50 ± 15.66	141.38 ± 22.36	0.192
DBP, mm Hg	75.06 ± 11.61	77.70 ± 12.78	0.476	80.00 ± 14.63	77.70 ± 12.78	0.697	71.10 ± 7.02	80.00 ± 14.63	0.146
Male sex, %	100	100	…	100	100	…	100	100	…
Statins, *n*	18	30	…	8	30	…	10	8	…
Antihypertensives, *n*	17	26	0.637	7	26	1.000	10	7	0.444
Insulins, *n*	6	0	0.002	4	0	< 0.001	2	4	0.321
Oral antidiabetic agents, *n*	12	0	< 0.001	2	0	0.040	10	2	0.002

	Study group 2 (targeted analysis)								
	T2DM *n* = 26	Non-DM *n* = 26	P	T2DM (− Met) *n* = 11	Non-DM *n* = 26	P	T2DM (+ Met) *n* = 15	T2DM (− Met) *n* = 11	P
	Mean ± SD	Mean ± SD		Mean ± SD	Mean ± SD		Mean ± SD	Mean ± SD	
Age at surgery, years	67.84 ± 4.83	68.99 ± 5.24	0.410	67.35 ± 4.21	68.99 ± 5.24	0.367	68.25 ± 5.35	67.35 ± 4.21	0.670
HbA1c, %	7.11 ± 1.11	5.60 ± 0.37	< 0.001	7.41 ± 1.39	5.60 ± 0.37	< 0.001	6.84 ± 0.75	7.41 ± 1.39	0.231
HbA1c, mmol/mol	54.24 ± 12.17	37.71 ± 4.03	< 0.001	57.48 ± 15.19	37.71 ± 4.03	< 0.001	51.28 ± 8.16	57.48 ± 15.19	0.230
Cholesterol, mmol/L	3.76 ± 1.04	4.20 ± 1.26	0.230	4.00 ± 1.32	4.20 ± 1.26	0.692	3.55 ± 0.68	4.00 ± 1.32	0.329
LDL, mmol/L	1.85 ± 0.69	2.33 ± 0.90	0.058	2.07 ± 0.87	2.33 ± 0.90	0.447	1.65 ± 0.42	2.07 ± 0.87	0.174
HDL, mmol/L	1.13 ± 0.24	1.41 ± 0.51	0.030	1.14 ± 0.25	1.41 ± 0.51	0.129	1.12 ± 0.25	1.14 ± 0.25	0.851
Triglycerides, mmol/L	1.90 ± 0.89	1.21 ± 0.35	0.002	1.89 ± 1.03	1.21 ± 0.35	0.011	1.91 ± 0.79	1.89 ± 1.03	0.960
Creatinine, µmol/L	96.12 ± 29.47	86.40 ± 16.93	0.160	95.64 ± 32.61	86.40 ± 16.93	0.269	96.47 ± 28.12	95.64 ± 32.61	0.945
BMI, kg/m^2^	28.66 ± 3.95	27.13 ± 3.69	0.150	27.21 ± 3.41	27.13 ± 3.69	0.949	29.73 ± 4.08	27.21 ± 3.41	0.110
SBP, mm Hg	141.12 ± 18.64	139.92 ± 19.79	0.820	138.82 ± 10.59	139.92 ± 19.79	0.863	142.80 ± 23.09	138.82 ± 10.59	0.601
DBP, mm Hg	74.92 ± 10.42	77.73 ± 10.71	0.340	75.73 ± 12.28	77.73 ± 10.71	0.621	74.33 ± 9.24	75.73 ± 12.28	0.744
Male sex, %	85	85	…	82	85	…	87	82	…
Statins, *n*	26	26	…	11	26	…	15	11	…
Antihypertensives, *n*	21	13	0.040	8	13	0.285	13	8	0.620
Insulins, *n*	6	0	0.023	3	0	0.021	3	3	0.218
Oral antidiabetic agents, *n*	19	0	< 0.001	4	0	0.005	15	4	< 0.001

For each study group three comparisons of clinical data are made: T2DM vs. non-DM patients; non-metformin-treated T2DM (− Met) vs. non-DM patients; and non-metformin-treated T2DM (− Met) vs. metformin-treated T2DM patients (+ Met). Results are shown as mean ± SD, percentage (%) or number (*n*) as indicated

In study group 1, one slide cracked during the hybridization process. Since no miRNA expression data is available for this sample, the accompanying patient data is not included in the table

*HbA1c* glycated hemoglobin, *LDL* low-density lipoprotein, *HDL* high-density lipoprotein, *BMI* Body-mass index, *SBP* systolic blood pressure, *DBP* diastolic blood pressure

### Explorative analysis

**Fig. 2:**
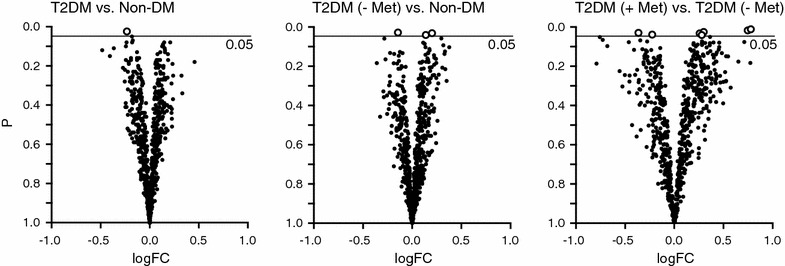
MiRNA expression profiling in arterial samples from T2DM and non-DM patients. MiRNA profiling was performed by microarray analysis. Data is represented as volcano plots for the following comparisons: T2DM vs. non-DM patients (reference group); non-metformin-treated T2DM (− Met) vs. non-DM patients (reference group); and non-metformin-treated T2DM (− Met) vs. metformin-treated T2DM patients (+ Met) (reference group). MiRNAs with P values less that 0.05 (prior to false discovery rate (FDR) correction of multiple testing) are represented as circles. The x-axis shows the log2 fold-change in miRNA expression and y-axis shows the *P* value

To screen for vascular miRNAs dysregulated in the setting of T2DM, RNA was purified from fresh frozen ITAs and analyzed by miRNA microarray. 677 probes were included in the analysis based on pre-defined criteria for probe signal intensity (see “Methods” section). Prior to correction for multiple testing, a single miRNA (miR-142-3p) was differentially expressed between T2DM and non-DM patients. Three miRNAs (miRPlus-B1114, miR-5584-3p, and miR-5006-3p) were differentially expressed comparing non-metformin-treated T2DM- and non-DM patients, and seven miRNAs (miR-491-3p, miRPlus-A1086, miR-193b-3p, miR-3935, miR-1252-5p, miRPlus-B1114, and miR-5681b) were differentially expressed between metformin-treated and non-metformin-treated T2DM patients (Fig. 2). However, no miRNAs were found to be affected post FDR correction.

### Targeted analysis

We then performed an independent experiment using a targeted qPCR-based approach. A panel of 12 miRNAs was selected (Table 2). This panel included miR-221-5p and miR-222-5p which were reported to be affected by diabetes in a similar setting by others [28]. We also included miR-29a-3p and miR-29b-3p—the primary candidates to regulate the level of α1- and α2-chain of collagen IV [12–17]. Finally, we included eight miRNAs (miR-21-5p, miR-24-3p, miR-26a-5p, miR-182-3p, miR-145-5p, miR-132-3p, miR-212-3 and miR-1298-5p) previously demonstrated to be involved in phenotypic modulation of vascular smooth muscle cells [29–37], since these are the vast majority of cells in ITAs.

**Table 2:** QPCR analysis of selected miRNAs in arterial FFPE samples from T2DM and Non-DM patients

microRNA	Assay nr.	References	T2DM *n* = 26	Non-DM *n* = 26	P	T2DM (− Met) *n* = 11	Non-DM *n* = 26	P	T2DM (+ Met) *n* = 15	T2DM (− Met) *n* = 11	P
			Mean ± SD	Mean ± SD		Mean ± SD	Mean ± SD		Mean ± SD	Mean ± SD	
miR-21-5p	397	[37]	0.112 ± 0.079	0.101 ± 0.098	0.928	− 0.012 ± 0.098	0.101 ± 0.098	0.493	0.204 ± 0.117	− 0.012 ± 0.090	0.183
miR-24-3p	402	[29, 30]	0.123 ± 0.093	0.100 ± 0.114	0.876	− 0.099 ± 0.114	0.100 ± 0.114	0.303	0.286 ± 0.126	− 0.099 ± 0.114	0.039
miR-26a-5p	405	[31]	0.120 ± 0.071	0.116 ± 0.096	0.978	− 0.018 ± 0.096	0.116 ± 0.096	0.408	0.220 ± 0.095	− 0.018 ± 0.095	0.097
miR-182-3p	483	[32]	1.215 ± 0.264 (*n* = 10)	1.449 ± 0.179 (*n* = 17)	0.456	1.037 ± 0.179 (*n* = 5)	1.449 ± 0.179 (*n* = 17)	0.326	1.393 ± 0.297 (*n* = 5)	1.037 ± 0.457 (*n* = 5)	0.532
miR-145-5p	2278	[33, 34]	0.134 ± 0.088	0.107 ± 0.109	0.846	− 0.067 ± 0.109	0.107 ± 0.109	0.346	0.281 ± 0.121	− 0.067 ± 0.103	0.047
miR-29a-3p	2112	[12–17]	0.158 ± 0.075	0.180 ± 0.084	0.849	0.064 ± 0.084	0.180 ± 0.084	0.434	0.227 ± 0.105	0.064 ± 0.104	0.294
miR-29b-3p	413	[12–17]	0.150 ± 0.091	0.220 ± 0.087	0.582	0.017 ± 0.087	0.220 ± 0.087	0.240	0.247 ± 0.102	0.017 ± 0.160	0.216
miR-221-5p	2096	[28]	NA	NA	…	NA	NA	…	NA	NA	…
miR-222-5p	2097	[28]	0.246 ± 0.118 (*n* = 25)	0.306 ± 0.084	0.674	− 0.135 ± 0.084 (*n* = 10)	0.306 ± 0.084	0.011	0.499 ± 0.134	− 0.135 ± 0.154 (*n* = 10)	0.006
miR-132-3p	457	[35]	0.123 ± 0.090	0.189 ± 0.105	0.636	− 0.040 ± 0.105	0.189 ± 0.105	0.231	0.242 ± 0.108	− 0.040 ± 0.144	0.122
miR-212-3p	515	[35]	− 0.072 ± 0.043	− 0.013 ± 0.075	0.501	− 0.122 ± 0.075	− 0.013 ± 0.075	0.358	− 0.035 ± 0.072	− 0.122 ± 0.028	0.326
miR-1298-5p	2861	[36]	− 0.099 ± 0.041	0.035 ± 0.070	0.104	− 0.222 ± 0.070	0.035 ± 0.070	0.031	− 0.009 ± 0.047	− 0.222 ± 0.054	0.007

For each of the 12 miRNAs three patient group comparisons were made: T2DM vs. non-DM patients; non-metformin-treated T2DM (− Met) vs. non-DM patients; and non-metformin-treated T2DM (− Met) vs. metformin-treated T2DM patients (+ Met). Normalization was performed using equal amounts of total RNA for each sample and by normalizing the relative expression to the synthetic miRNA ath-miR-159a. Values are mean ± SD. Assay nr. refers to TaqMan microRNA assays (Life Technologies)

Important for the aim of our study, the RNA used for this analysis was obtained from sections of the same FFPE tissue that we used to document elevated levels of basement membrane proteins [8, 10].

Although most miRNAs were successfully quantified in all samples, we failed to detect miR-182-3p in 25 of 52 samples and miR-221-5p in all of the 52 samples. We assume this is due to low abundance since both miR-182-3p- and miR-221-5p were excluded from the microarray analysis, because of low signal intensity, however technical issues could potentially also be an issue.

Nominal p-values below 0.05 were found for several miRNAs in the intergroup comparisons: miR-222-5p and miR-1298-5p [T2DM (− Met) vs. non-DM], and miR-24-3p, miR-145-5p, miR-222-5p and miR-1298-5p [T2DM (+ Met) vs. T2DM (− Met)] (Table 2). However, following Bonferroni correction of multiple testing no significant differences were observed. An interesting pattern is observed for miR-222-5p and miR1298-5p as expression is reduced in non-metformin treated T2DM patients as compared to both non-DM patients and metformin-treated patients, suggesting that metformin treatment counteracts the effect of T2DM. However, the same trends were not seen in the microarray data (Additional file 1).

## Discussion

To the best of our knowledge, this is the first study to assess the influence of diabetes on macrovascular miRNA expression using an explorative approach. Unexpectedly, we were unable to detect any vascular miRNAs for which the expression level is affected by either T2DM status or metformin-treatment. Moreover, we were not able to detect differential expression of 12 selected miRNAs using a targeted qPCR-based approach in an independent study group, which we previously have used to demonstrate distinct diabetes-induced changes at the protein level [8].

This finding is not consistent with a previous report by Coleman et al. showing a more than twofold elevation in the expression of miR-221-5p and miR-222-5p in ITAs from non-metformin-treated T2DM patients as compared to both non-DM patients and metformin-treated T2DM patients [28]. miR-221-5p was excluded from the microarray analysis as it did not reach the threshold of signal intensity, and was not detectable by qPCR in any sample of study group 2. miR-222-5p was detected in both analyses, yet we found no significant effect of T2DM or metformin-treatment. The explanation underlying this discrepancy is unclear, however, while study populations are comparable in size, differences do exist. In Coleman et al., the T2DM patients that were not treated with metformin are obese (BMI: 34.8 ± 1.4), while the equivalent group in both our study groups are merely overweight [BMI: 29.2 ± 2.6 (Study group 1) and 27.2 ± 3.4 (Study group 2)], which may explain dissimilarities, since obesity is closely related to epigenetic alterations [38]. Importantly, technical variability caused by differences in tissue preparation, RNA purification, reverse transcription, qPCR methodology and/or normalization strategies may contribute to the observed differences between the study by Coleman et al., and this study.

Evaluation of changes induced specifically by diabetes (being hyperglycemia or vascular insulin resistance) on vascular cells and tissue that might prime subsequent pathological mechanisms necessitates analysis of non-diseased artery tissue. Our study focus on such putative alterations and is based on analysis of ITAs devoid of lesions. The microRNAome is strongly affected by artery lesions such as intimal hyperplasia or atherosclerosis [39, 40] and the presence of such lesions could potentially outweigh the influence of diabetes on vascular cells. Moreover, varying degree of disease progression between samples would introduce heterogeneity and intragroup variability, which reduce the ability to detect intergroup differences. As we base our study on non-lesional arteries we do not address potential effects of diabetes on miRNA expression after lesion initiation. Although difficult to detect due to the reasons stated above, hyperglycemia or vascular insulin resistance might have an effect on miRNA expression of inflammatory cells or phenotypically modulated artery cells. Also, diabetes may affect miRNA expression in the presence of other pathological stimuli like inflammation. Since the purpose of our study is to identify diabetes-induced vascular changes that precede and prime pathological processes, it is a limitation that we use an artery that is lesion resistant. However, vascular alterations induced by e.g. hyperglycemia presumably affects all arteries similarly, while the principal determinant of the susceptibility to atherosclerosis is local hemodynamics [41].

After an initial explorative analysis, we focus on a panel of 12 miRNAs in the targeted analysis. The 12 miRNAs selected for this analysis have previously been reported to be regulated in the setting of diabetes, to be known regulators of collagen IV expression, or to affect phenotypic modulation of vascular smooth muscle cells. Not considering miRNAs implicated in other vascular disease processes such as inflammation [42] or other diabetes-related conditions [43–45] in the targeted analysis is a limitation in our study. Moreover, several factors and pathways are known to be associated to vascular complications in diabetic patients, and since we do not have comprehensive data whether our patients display these characteristics, we cannot exclude that differences in such factors may have influenced our results.

We previously identified elevated levels of a distinct group of proteins associated with basement membranes in non-lesional arteries of T2DM patients. The underlying mechanisms remain elusive, although it does not appear to be associated with altered expression of the genes encoding these proteins. In this study we sought to identify whether miRNA expression was affected by T2DM status, and if such regulation could explain our previous observations. As we were unable to detect differential expression of miRNAs between T2DM and non-DM patients, we suggest that other mechanisms of regulation such as decreased protein degradation in diabetics underlie our previous findings.

## Conclusions

Our data suggest that alternatives to microRNA dysregulation underlie T2DM-associated protein changes in non-atherosclerotic arteries.

## Additional file

**Additional file 1.** MicroRNA microarray results. MicroRNA microarray data analysis for 677 probes comparing (1) T2DM vs. non-DM patients, (2) T2DM (− Met) vs. non-DM patients, and (3) T2DM (+ Met) vs. T2DM (− Met).

## Abbreviations

CVD: cardiovascular disease
ITA: internal thoracic artery
CABG: coronary artery bypass grafting
T2DM: type 2 diabetes mellitus
non-DM: non diabetes mellitus
miRNA: microRNA
